# The overlooked role of microbiota-gut-brain communication in child psychiatry: a call for integration in early intervention strategies

**DOI:** 10.1080/19420889.2024.2446332

**Published:** 2025-01-02

**Authors:** Sunny Cui, Mubtaseem Aronno, Angel K.Q. Wong, Leah Snodgrass

**Affiliations:** a Department of Biological Sciences, Dartmouth College, Hanover, NH, USA; b Schulich School of Medicine and Dentistry, Western University, London, ON, Canada; c Department of Psychiatry, DeBusk College of Osteopathic Medicine, Harrogate, TN, USA

**Keywords:** microbiota-gut-brain axis, Child psychiatry, neuroimmune interactions, microbiota communication, neurodevelopmental disorders

## Abstract

Emerging research has highlighted the significant role of microbiota-gut-brain communication in child psychiatric disorders, including autism spectrum disorder (ASD) and anxiety disorders. Despite this, mainstream psychiatric interventions for children continue to focus predominantly on neurological and psychological therapies, neglecting the critical influence of gut microbiota on brain development and behavior. This commentary underscores the need for greater integration of microbiota-targeted therapies, such as dietary interventions, prebiotics, and probiotics, into early psychiatric intervention strategies. By addressing the gut-brain axis as a key component of neurodevelopmental and psychiatric outcomes, clinicians can adopt a more holistic and biologically informed approach to treatment. We propose that future research and clinical practice should prioritize interdisciplinary collaboration to explore how microbiota-based treatments can be incorporated into existing child psychiatry frameworks, offering new avenues for improving long-term mental health outcomes.

## Introduction

Child psychiatric disorders such as autism spectrum disorder (ASD), mood disorders, and attention-deficit/hyperactivity disorder (ADHD) have become increasingly prevalent, and have significant implications for both patients and healthcare systems [1]. However, despite advances in our understanding of neurodevelopmental and psychiatric disorders, the treatment landscape mostly centers on psychopharmacological and behavioral therapies. While effective, these approaches often overlook a growing body of evidence linking gut microbiota dysbiosis to mental health and developmental outcomes [2]. The microbiota-gut-brain axis, a bi-directional communication network between the gastrointestinal (GI) tract and the central nervous system (CNS), is emerging as a critical factor in modulating brain development, emotional regulation, and behavior.

In this commentary, we argue for the gradual integration of microbiota-targeted interventions, including probiotics, prebiotics, and dietary modifications, into early child psychiatric treatments. By addressing the microbiota-gut-brain axis alongside conventional therapies, clinicians could adopt a more holistic and biologically informed approach to improving long-term mental health outcomes. Novel avenues for enhancing the efficacy of treatment protocols, particularly in disorders with strong neurobiological underpinnings like ASD and ADHD, may be forthcoming as a result.

## The science of microbiota-gut-brain communication

The microbiota-gut-brain axis is mediated through neural, endocrine, immune, and metabolic pathways [3]. The vagus nerve, one of the primary conduits of gut-brain communication, allows direct neural transmission of signals from the gut to the CNS. Additionally, gut bacteria produce metabolites such as short-chain fatty acids (SCFAs), including butyrate, propionate, and acetate, which can cross the blood-brain barrier and influence neuroinflammatory processes and neurogenesis [4].

Key components of this communication network involve not only the neural circuits but also the hypothalamic–pituitary–adrenal (HPA) axis, a central player in stress regulation [5]. Gut dysbiosis has been shown to activate the HPA axis, leading to dysregulated cortisol production and increased stress responses, both of which are implicated in anxiety and mood disorders. Moreover, microbiota influence the production of neurotransmitters like serotonin, gamma-aminobutyric acid (GABA), and dopamine. It is estimated that up to 95% of serotonin, a key neurotransmitter in mood disorders, is produced in the gut [6].

Recent studies have demonstrated that alterations in the gut microbiome composition are associated with various psychiatric disorders in children. For example, children with ASD often exhibit gut microbiota dysbiosis, characterized by reduced diversity and an overabundance of certain bacterial species like *Clostridia* and *Bacteroidetes*, known to produce neuroactive metabolites [7]. Similarly, alterations in the gut microbiota have been linked to anxiety disorders, with specific taxa like *Lactobacillus, Bifidobacterium*, and *Faecal bacterium* playing roles in modulating anxiety behavior in animal models [8].

In the early stages of life, factors such as mode of delivery (vaginal vs. cesarean), breastfeeding, antibiotic use, and diet, play crucial roles in shaping the gut microbiota, which in turn influences brain development [9–11]. Early-life disruptions to the microbiota, particularly during the critical window of neurodevelopment, may predispose children to neurodevelopmental disorders by affecting neuroimmune and neuroendocrine signaling pathways. Animal studies have provided robust evidence that germ-free mice exhibit increased stress responses, cognitive deficits, and abnormal social behaviors, all of which are partially restored by the introduction of specific microbiota strains [12].

## The gap in current child psychiatry practices

Despite mounting evidence of the microbiota-gut-brain axis’s role in psychiatric outcomes, current child psychiatry practices remain predominantly focused on neurological and psychological therapies. Traditional interventions often emphasize pharmacological treatments targeting neurotransmitter systems, such as selective serotonin reuptake inhibitors (SSRIs) for anxiety and depression, antipsychotics for ASD-related irritability and/or aggression, and stimulants such as amphetamines for ADHD. Cognitive-behavioral therapy (CBT) is a ubiquitous psychotherapeutic recommendation which, combined with medication, is a mainstay of child psychiatric care. However, continued neglect of the gut-brain axis may limit our ability to treat the full spectrum of psychiatric disorders, particularly in cases where gut dysbiosis is involved. Furthermore, given the early-life influence of gut microbiota on brain development, incorporating microbiota-targeted therapies into early interventions may enhance neurodevelopmental trajectories and reduce the severity of psychiatric symptoms over time.

## Potential integration of microbiota-based therapies

Given the significant role of the microbiota-gut-brain axis in mental health, there is growing interest in developing microbiota-based interventions as adjunctive therapies for children with various psychiatric disorders. Probiotics, live microorganisms that provide health benefits when consumed in adequate amounts, have shown promise in modulating the microbiota-gut-brain axis and improving psychiatric outcomes, particularly in children with neurodevelopmental disorders. A 2023 review by Feng et al. analyzed 15 clinical studies assessing probiotic supplementation in children with autism spectrum disorder (ASD) and found general improvements in behavioral scores and ASD-related symptoms [13]. Similarly, a 2020 double-blind, placebo-controlled pilot trial exploring the effects of the probiotic, *Lactobacillus rhamnosus GG* (ATCC 53,103), on ADHD, found that after three months of treatment, children receiving the probiotic demonstrated significant improvements in their PedsQL Child Self-Report Total Score (*p* = .021, d = 0.53) [14]. Additionally, Pärtty et al. followed infants who received *L. rhamnosus GG* during the first six months of life for 13 years, finding that none of the probiotic group developed ADHD or Asperger syndrome (AS) by age 13, compared to a 17.1% diagnosis rate in the placebo group (*p* = .008) [15]. These findings suggest that early probiotic supplementation may play a protective role against the development of neurodevelopmental disorders. Although large-scale trials in children with mood and depressive disorders are limited, clinical studies in adults have found reduced cortisol and depressive symptoms with daily intake of probiotics [16].

Another method of modulating the gut microbiota is prebiotics, non-digestible fibers that promote the growth of beneficial gut bacteria, sometimes used in conjunction with probiotics. Dandekar et al. investigated the effects of a multi-strain probiotic formulation, Cognisol, on rats exposed to maternal separation and chronic unpredictable mild stress, simulating anxiety- and depression-like symptoms [17]. Probiotic supplementation significantly reversed stress-induced behavioral abnormalities, including anhedonia, reduced mobility, and anxiety-like behavior, alongside the restoration of serotonin levels. In human studies, Schmidt et al. conducted a randomized controlled trial in adults, where prebiotic B-GOS intake significantly reduced salivary cortisol awakening response, a key marker of stress, compared to placebo [18]. In children, Palmer et al. [19] demonstrated in a 2024 randomized, double-blind, placebo-controlled trial that prebiotic B-GOS supplementation significantly improved social behavior in children with ASD. Furthermore, Yang et al. [20] showed that Synbiotic 2000, a combination of prebiotics, postbiotics, and short-chain fatty acids (SCFAs), reduced pro-neuroinflammatory markers like IL-12/IL-23p40 and sICAM-1 in children with.

Adjunctive dietary interventions have also shown promise in improving mental health. In a 12-week randomized controlled trial, Jacka et al. [21] found that adults with major depressive disorder (MDD) who received nutritional consulting had significantly greater improvement on the Montgomery–Åsberg Depression Rating Scale (MADRS) compared to controls (*p* < .001, Cohen’s d = −1.16). Similarly, a 2022 systematic review by Wobido et al. [22] assessed omega-3 supplementation in children with ASD, showing a modest improvement in ASD symptoms (SMD = −0.13; CI 95% = −0.34, −0.02) [22]. However, subgroup analyses showed inconsistent efficacy. Given the variability in dietary interventions, more standardized studies are needed to fully assess their impact on mental health and neurodevelopmental disorders in children.

Fecal microbiota transplantation may also offer a promising approach, where a healthy donor stool is transplanted into the gut of a recipient to restore a balanced gut microbiome. Recent reviews have shown that it may be beneficial in alleviating symptoms of depression, ADHD, ASD, and various other mood disorders [7,23–25].

Practical challenges in integrating microbiota-based therapies into clinical practice exist. Individual variations in microbiota composition, influenced by genetics, diet, and environment, necessitate a personalized approach to treatment. Moreover, while early-stage research is promising, larger-scale, randomized controlled trials specific to a pediatric population are needed to establish the efficacy and safety of microbiota-targeted interventions in children.

## Call for a holistic approach

The integration of microbiota-targeted therapies into child psychiatry requires an interdisciplinary approach involving collaboration between psychiatrists, microbiologists, nutritionists, and immunologists. This holistic approach may offer a more comprehensive solution to child psychiatric disorders, particularly those thought to be linked to microbiota-gut-brain axis like ASD, ADHD, and anxiety, when found to be resistant to traditional therapies. For instance, interventions could include pairing probiotics with specific nutrient-rich diets tailored to an individual’s microbiota composition or incorporating prebiotic intake as part of routine therapy.

## Conclusion

The microbiota-gut-brain axis represents a critical but underexplored factor in child psychiatry. By integrating microbiota-targeted therapies into early interventions, clinicians may improve the long-term mental health outcomes of children with psychiatric disorders. The emerging evidence underscores the importance of a holistic, biologically informed approach to treatment, one that considers the complex interplay between the gut and brain in shaping behavior, cognition, and emotional regulation.

## Data Availability

As new data was not generated or analyzed in this study, data sharing is not applicable.
